# Endoplasmic Reticulum Stress Induces miR-706, A Pro-Cell Death microRNA, in A Protein Kinase RNA-Like ER Kinase (PERK) and Activating Transcription Factor 4 (ATF4) Dependent Manner 

**DOI:** 10.22074/cellj.2020.6873

**Published:** 2019-12-15

**Authors:** Xiu Wang, Yi Han, Guodong Hu, Jianbo Guo, Hongyu Chen

**Affiliations:** 1 Department of Anesthesiology, The Fourth Affiliated Hospital of China Medical University, Shenyang, Liaoning, China; 2 The Second Department of Urology, Shenyang Red Cross Hospital, Shenyang, People’s Republic of China (CHN); 3 The Third Department of General Surgery, The Fourth affiliated Hospital of China, Medical University, Shenyang, Liaoning, China

**Keywords:** Activating Transcription Factor 4, Caspase Activity and Apoptosis Inhibitor 1, Endoplasmic Reticulum Stress, miR-706, Protein Kinase RNA-Like ER Kinase

## Abstract

**Objective:**

Endoplasmic reticulum (ER) stress causes an adaptive response initiated by protein kinase RNA-like ER kinase (PERK), Ire1 and ATF6. It has been reported that these upstream regulators induce microRNAs. The current study was designed to find a novel microRNA that mediates ER stress components and finally contributes to cell fate decision.

**Materials and Methods:**

In this experimental study, miR-706 levels were checked under different conditions of ER stress induced by Thapsigargin, Tunicamycin or low glucose media. PERK and ATF4 were knocked-down by administration of lentivirus-mediated short hairpin RNA to explore the effect of ER stress related proteins on miR-706 expression. The effect of miR-706 on caspase activity and apoptosis inhibitor 1 (CAAP1) levels were examined by using mimic-miR-706. The role of CAAP1 in inhibiting cell death (measured by Annexin V staining) and contributing to patient overall survival (measured by Kaplan-Meier estimate) were further confirmed by anti- miR-706 and CAAP1 knock-down.

**Results:**

We showed that Thapsigargin or Tunicamycin triggered ER stress leading to the induction of miR-706. miR-706 induction is dependent on PERK and its downstream regulator ATF4, as knocking-down of PERK and ATF4 suppressed miR-706 induction in response to ER stress. Knocking-down of miR-706 reduces cell death triggered by ER stress, indicating that miR-706 is pro-cell death microRNA. We further identified CAAP1 as a miR-706 target in regulating ER stress initiated cell death.

**Conclusion:**

Collectively, our results pointed to an ER signaling network consisting of proteins, microRNA and novel target.

## Introduction

Unfolded protein response (UPR) or endoplasmic reticulum (ER) stress is a signaling pathway elicited in response to various stimuli such as hypoxia during low oxygen pressure in tumors, virus infection and other stresses which disturb cell homeostasis (1-3). During UPR, unfolded proteins are accumulated in the ER lumen and these then interact with binding immunoglobulin protein (BiP, also known as GRP- 78) (4-6). In homeostatic cell, BiP is bound to three upstream regulators of UPR, as protein kinase RNAlike ER kinases (PERKs), IRE1 and ATF6 (7, 8). In the presence of unfolded proteins, BiP is released from these UPR regulators and binds to unfolded proteins. This triggers activation of PERK, Ire1 and ATF6 by oligomerization (9, 10). Ire1 is composed of a domain that senses stress and it is in the lumen of the ER. This protein has a single transmembrane domain as well as the cytosolic domain that contains a protein kinase sub-domain and RNase sub-domain (11). Ire1 triggers expression of X-box binding protein 1 (Xbp1) transcription factor (12).

PERK activation leads to translational suppression via eIF2 alpha serine 51 phosphorylation and this causes ATF4 production (13). ATF4 is a transcription factor which in turn induces pro-apoptotic protein CHOP (14-17). PERK plays important role in diabetes, cancer and Alzheimer’s disease (14, 18-20). PERK has dual seemingly contradictory activities. When the stress is brief, PERK induces anti-apoptotic components like miR-211, while the stress is prolonged, it induces proapoptotic CHOP (16).

microRNAs (miR) are almost 22 nucleotides RNAs coding no protein. However, they perform diverse functions in a variety of biological processes by posttranscriptional regulation of gene expression (21-24). Regulation of microRNAs by all three branches of UPR (PERK, IRE1 and ATF6) has been well documented in the past decades (25, 26). Here, we show that PERK induces miR-706 and this microRNA promotes cell death in the later stages of cell stress. The miR-706 dependent cell death is regulated by the PERK/ATF4 signaling through targeting caspase activity and apoptosis inhibitor 1 (CAAP1).

## Materials and Methods

### Cell cultures

In this experimental study, NIH3T3 mouse embryonic fibroblasts (CRL1658, ATCC, USA) and AML12 mouse hepatic cells (CRL-2254™, ATCC) were cultured in Dulbecco’s Modified Eagle Medium (DMEM, Invitrogen, USA) supplemented with 10% fetal bovine serum (Sigma-Aldrich, USA) at 37˚C in a humidified atmosphere of 5% CO2. The cells were passaged every two days at 1:6 ratios. The cells were treated with 500 nM Thapsigargin (Sigma-Aldrich, USA) or 100 ng/ml Tunicamycin (Sigma-Aldrich, USA) unless otherwise noted. Human lung cancer cell line H1299 (CRL5803, ATCC), human ovarian cancer line SKOV3 (HTB-77, ATCC), human ovarian surface epithelial cell line HOSE (Beinachuanglian, PRC) and human lung fibroblast HFL1 (CCL153, ATCC) cells were cultured in RPMI 1640 (Invitrogen, USA) with 10% fetal bovine serum.

### Knocking-down of PERK, ATF4 and CAAP1

Short hairpin RNA targeting PERK, ATF4 or CAAP1 from Dharmacon (USA) was transfected together with packaging plasmids (respectively pMDL, pVSVG or pRSV-Rev) into 293T cells by Lipofectamine 2000 (ThermoFisher, USA). For lentiviral transduction, NIH3T3 cells were incubated with 2 ml virus and 10 μg/ml polybrene (Sigma-Aldrich, USA) for three hours (27).

### Mimic and antisense miR-706

Mimic miR-706 (MmiR3117-MR03) was purchased from Genecopoeia (USA). Oligos targeting miR-706 (MmiR-AN1135-SN-20, Genecoepoeia) were transfected using Lipofectamine 2000 as previously reported (28).

### Quantitative reverse transcription polymerase chain reaction and Western blot

Ambion microRNA purification kit (Ambion, USA) and microRNA reverse transcription kit (Applied Biosystems, USA) were used for total RNA preparation and RNA reverse transcription. Quantitative reverse transcription PCR (qRT-PCR) was performed on an Applied Biosystems 7900 apparatus (Applied Biosystems, USA). Primers for miR-706, ATF4, and CHOP included:

miR-706- F: 5´-ACACTCCAGCTGGGACAGAAACCCTGTCTC-3´
  R: 5´-TGGTGTCGTGGAGTCG-3´
ATF4- F: 5´-TCCTGAACAGCGAAGTGTTG-3´
  R: 5´-ACCCATGAGGTTTCAAGTGC-3´
CHOP- F: 5´-CTGCCTTTCACCTTGGAGAC-3´
  R: 5´-CGTTTCCTGGGGATGAGATA-3´.

Western blot was also conducted as previously reported (29). The antibodies are: CAAP1 (NBP1-94020, Novus Biologicals, USA) and GAPDH (14C10, Cell Signaling Technology, USA).

### Measurement of cell death

Cells were trypsinized, spun and stained with APCannexin V (30) (BD Biosciences, USA) according to the manufacturer’s protocol. The samples were run on FACS Canto (BD Biosciences, USA) to collect APC-annexin V fluorescence (31).

## Results

### miR-706 is an endoplasmic reticulum stress-dependent microRNA

**Fig 1:**
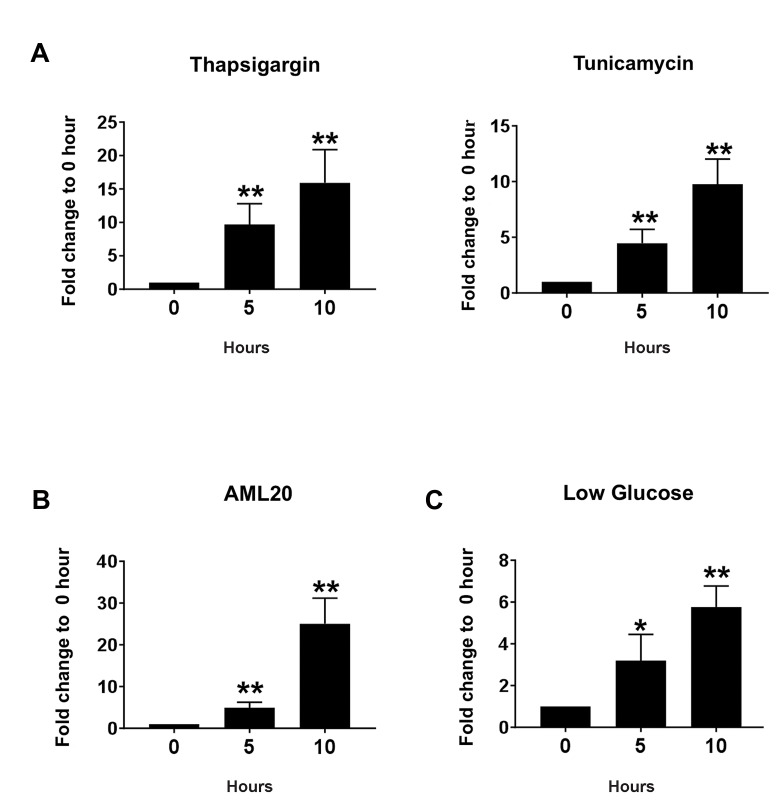
ER stress induces miR-706. **A.** NIH3T3 cells were treated with Thapsigargin or Tunicamycin at the indicated times. miR-706 expression was determnied by qRT-PCR. **B.** AML12 mouse hepatic cells were treated with Thapsigargin at the indicated times. miR-706 expression was determnied by qRT-PCR. C. NIH3T3 cells were put in low glucose media for the indicated times. miR-706 expression was determnied by qRT-PCR. *; P<0.05, **; P<0.01 compared to the control, and qRT-PCR; Quantitative reverse transcription polymerase chain reaction.

To determine if ER stress triggers miR-706, NIH3T3 cells were incubated with Thapsigargin for 5 and 10 hours. We used these time points as they are early enough to induce in full gear pro-apoptotic machinery (23, 24). Total RNA was purified and qRT-PCR analysis was done using miR-706 primers. We observed that in NIH3T3 cells, miR-706 expression was elevated in response to ER stress (Fig .1A, left panel). To confirm this result, NIH3T3 cells were further treated with Tunicamycin which has different mechanism of ER stress induction than Thapsigargin. Compared to the control cells, Tunicamycin treated cells showed significant extent amount of miR-706 induction (Fig .1A, right panel). These results imply that miR-706 is an ER stress-responsive microRNA.

We next treated hepatic cell line AML12 with Thapsigargin for ER stress induction. In these cells, there was also remarkable elevation of miR-706 expression (Fig .1B). To check whether physiological stimulus induces this microRNA, NIH3T3 cells were cultured in media with low glucose concentration (0.5 mM). We observed induction of miR-706 in low glucose media (Fig .1C). These findings confirmed that ER stress induces expression of miR-706.

### miR-706 is a PERK-dependent microRNA

**Fig 2:**
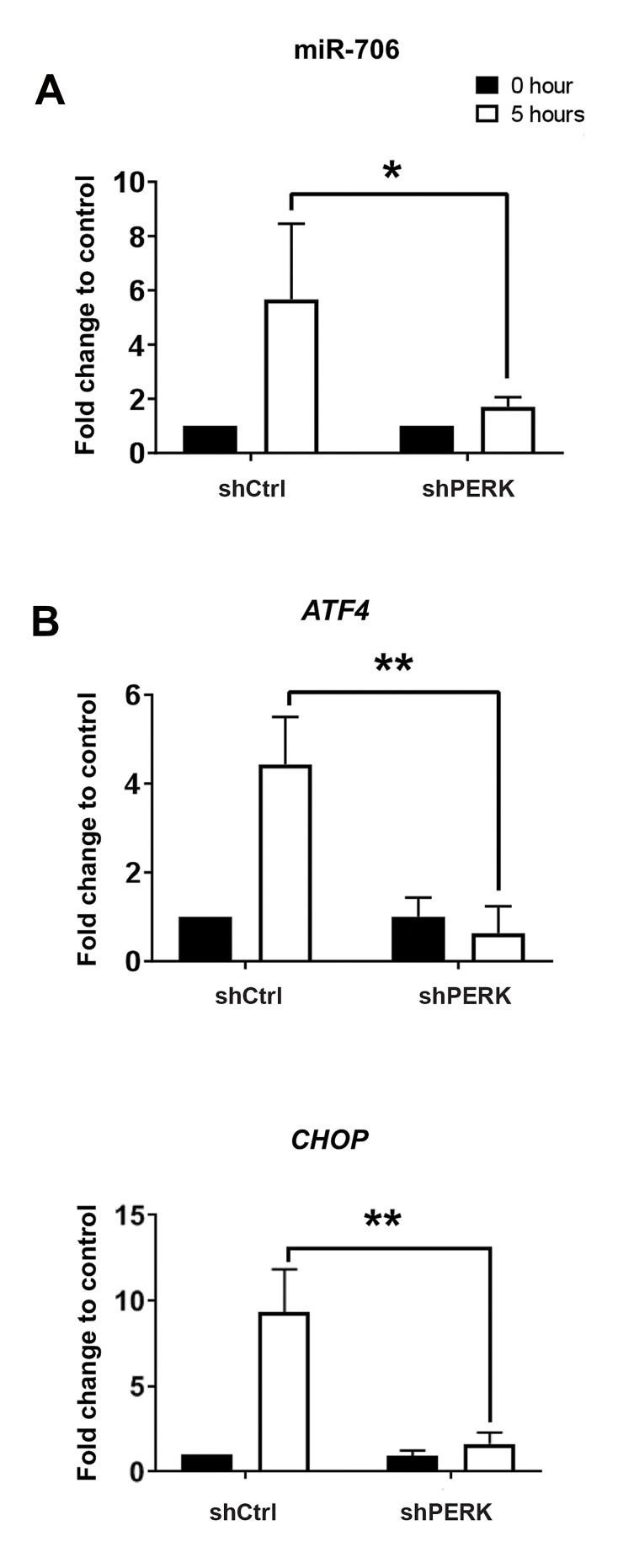
miR-706 induction in response to ER stress is dependent on PERK. NIH3T3 cells tranfected with shControl (shCtrl) or shPERK were treated with Thapsigargin for five hours. **A. **miR-706 expression level was analyzed by qRT-PCR. **B.** ATF4 and CHOP expression were measured by qRT-PCR as a read-out for PERK and ATF4 activity. *; P<0.05, **; P<0.01, and qRT-PCR; Quantitative reverse transcription polymerase chain reaction.

Next, we investigated if miR-706 induction by ERstress is PERK dependent. PERK wild-type and knockdown NIH3T3 cells were cultured and treated with Thapsigargin for 10 hours. Total RNA was purified from PERK wild-type and PERK knock-down cells. qRT-PCR using the specific primers showed that miR-706 was up-regulated 5.7 fold in PERK wild-type cells; this induction was remarkably suppressed in PERK knocked-down cells (Fig .2A). Since the activation of PERK leads to ATF4 translation inhibition and *CHOP* transcription (1), activity of PERK knock-down in our study was confirmed by assessing grade of downregulation of *ATF4* and *CHOP* transcriptions (Fig .2B). Knock-down of other branches of ER stress (IRE1 and ATF6) did not have significant effect on miR-706 induction (data not shown). These results demonstrate that miR-706 is governed in a PERK-dependent way in response to ER stress.

### miR-706 is an ATF4-dependent microRNA

**Fig 3:**
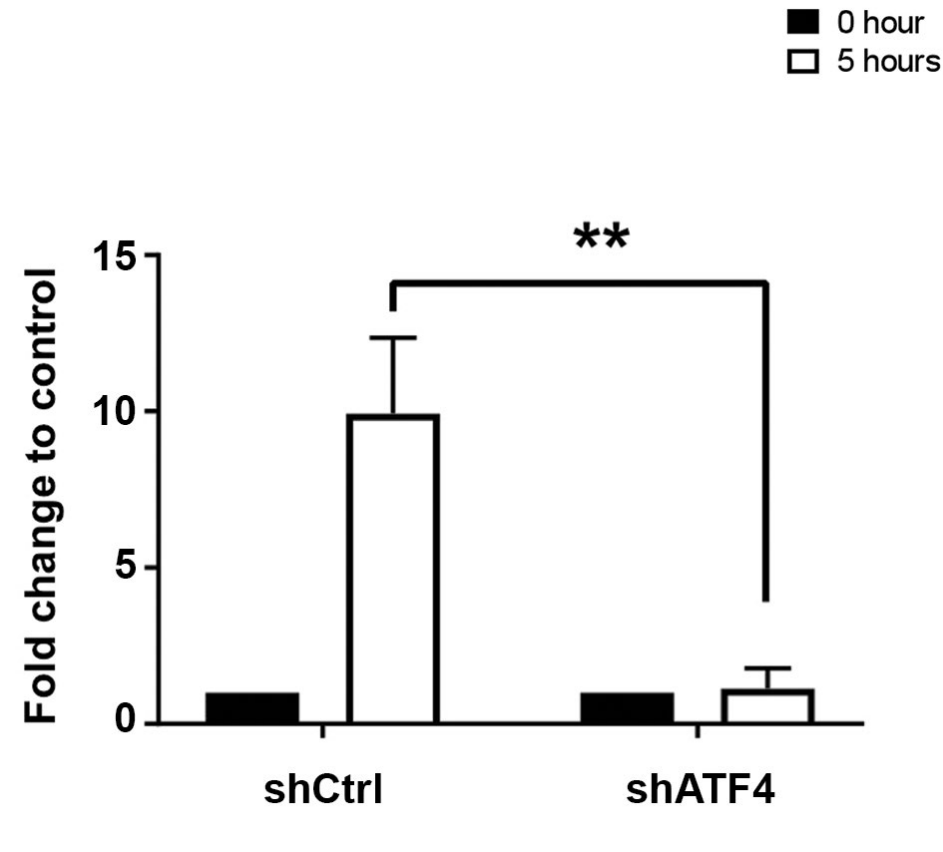
ATF is necessary for miR-706 induction in response to ER stress. ATF4 was knocked-down in NIH3T3 cells and treated with Thapsigargin for five hours. miR-706 expression were determnied by qRT-PCR . **; P<0.01 and qRT-PCR; Quantitative reverse transcription polymerase chain reaction.

ATF4 regulates transcription of many pro-survival and in case of prolonged stress, pro-apoptotic proteins like CHOP (32). To investigate downstream regulation of miR-706 synthesis, we treated ATF4 wild-type and ATF4 knock-down NIH3T3 cells with Thapsigargin for 0 and 10 hours. miR-706 expression was induced in ATF4 wild-type cells. ATF4 knock-down cells did not show any induction of miR-706 expression (Fig .3). These findings illustrate the crucial role of ATF4 in the ER/ miR-706 axis.

### miR-706 directly targets CAAP1

**Fig 4:**
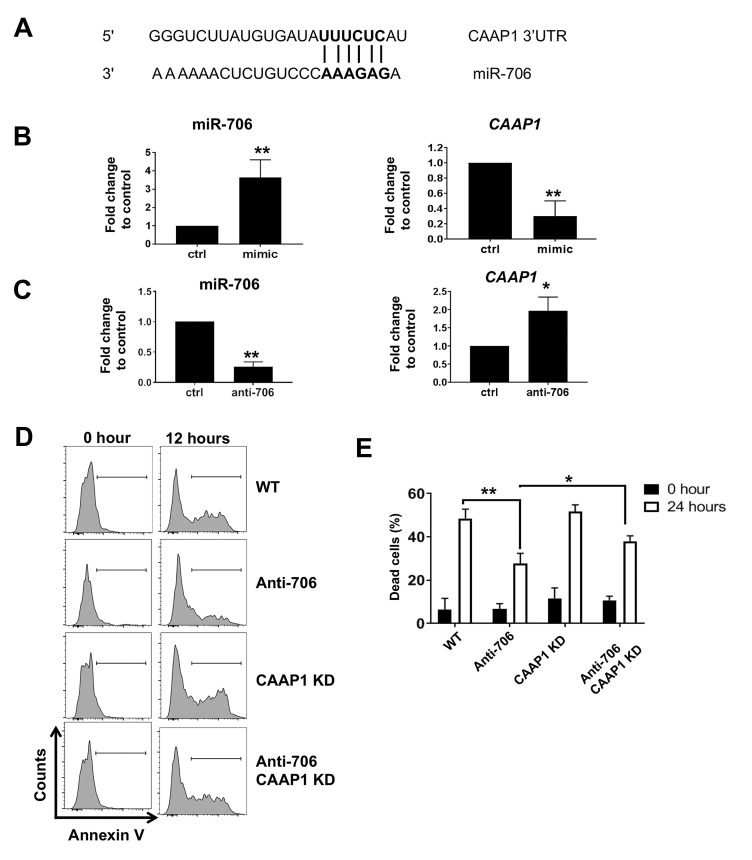
miR-706 tiggers ER stress dependent cell death trough CAAP1. **A.** Target scan prediction showing predicted binding of miR-706 and CAAP1 3ˊUTR. **B.** NIH3T3 cells were transfected with scrambled microRNA (control; ctrl) or mimic miR-706 (mimic). Total RNA was purified and CAAP1 expression was determined using qRT-PCR. **C.** NIH3T3 cells were transduced with antisense miR-706 (anti-706) and then incubated with Thapsigargin for 24 hours. Total RNA was purified and CAAP1 expression was determined using qRT-PCR. **D.** NIH3T3 cells were transduced with scrambled microRNA (ctrl), antisense miR-706 (anti-706) or antisense miR-706 and shCAAP1. The cells were then incubated with Thapsigargin for 24 hours. Cell death was assesed by Annexin V staining. **E.** Quantification of panel C. *; P<0.05, **; P<0.01 compared to control, and qRT-PCR; Quantitative reverse transcription polymerase chain reaction.

Using Targetscan analysis, microRNA target prediction showed that miR-706 is predicted to target anti-apoptotic caspase activity and apoptosis inhibitor 1 (CAAP1) (Fig .4A). Therefore, we used mimic miR-706 to overexpress miR-706 and then determine CAAP1 level. Figure 4B demonstrated that CAAP1 expression was attenuated in response to miR-706 overexpression. In addition, we checked the *CAAP1* level in antisense miR- 706 transduced cells. Figure 4C indicated down-regulation of *CAAP1* expression by anti- miR-706 in Thapsigargin treated cells. These data supported our hypothesis that CAAP1 is a miR-706 target.

### miR-706 promotes cell death following ER stress through CAAP1

Since miR-706 is induced by PERK and the latter molecule has dual pro-survival as well as pro-apoptotic functions (23), we studied whether miR-706 plays any role in cell fate. An oligo targeting miR-706 was designed and NIH3T3 cells were transduced. These cells were then treated with Thapsigargin for 24 hours. As a control, scrambled oligo was used. Total cell death in the scrambled and antisense miR-706 transduced cells was detected using Annexin V staining. We observed that compared to the scrambled transfected cells, cell death in the antisense miR-706 transduced cells was remarkably reduced, indicating that miR-706 is a pro-cell death microRNA. The cells in which miR-706 was inhibited and CAAP1 was knocked-down showed significant, even if partial, reversal of antisense miR-706 mediated protection (Fig .4D, E). These results indicated the fundamental role of CAAP1 in mediating miR-706 pro-apoptotic functions.

**Fig 5:**
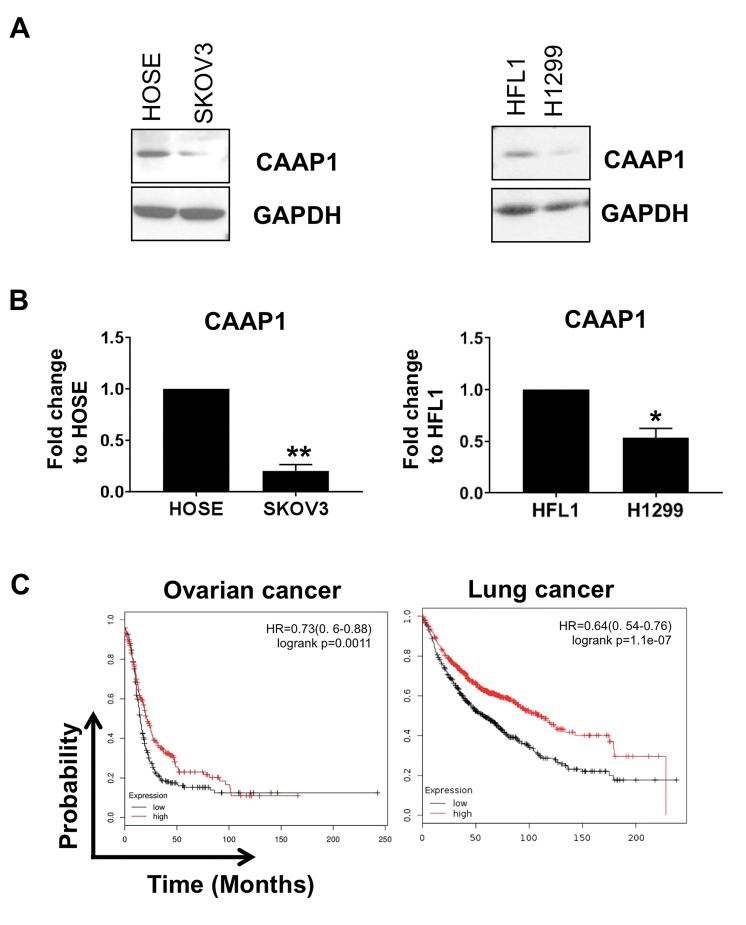
CAAP1 is critical for cell survival in tumor conditions that induce ER stress. **A.** CAAP1 protein levels are higher in the ovarian and lung cancer cell lines (SKOV3 and H1299) compared to their normal control cell lines (HOSE and HFL1). **B.** Quantification of panel A. *; P<0.05 or **; P<0.01 compared to HOSE or HFL1 cells respectively. **C.** High CAAP1 levels confer better survival in gastric, ovarian and lung cancer patients (analysis from the online database http:// kmplot.com/). Log-rank P values and hazard ratios are shown at the up right corner.

## Discussion

UPR or UPR has been shown to regulate several microRNAs. PERK up-regulates miR-30-c-2* which in turn represses Xbp-1. CHOP (36), as a downstream activator of PERK, induces miR-708, which in turn suppresses rhodopsin (25). PERK also induces miR-216b in a CHOPdependent manner, while it suppresses c-Jun and thereby induces apoptosis (24) . Ire1 has been shown to degrade premicroRNAs -125b and -96 with implications for cell death (26). In this work, for the first time, we identified a novel microRNA, miR-706, in response to ER stress and confirmed its critical role in governing cell fate.

ER stress signaling has dual purposes. In the initial stage, UPR signaling tries to preserve homeostasis by suppressing protein synthesis, launching antioxidant response and inducing pro-survival signals such as miR-211 (23). In the later stage, when it is clear that ER stress has caused damage beyond repair, UPR switches to pro-apoptotic signaling. Pro-apoptotic signals include CHOP. Interestingly, both pro-apoptotic and pro-survival signals can be initiated by the same transcription factors. ATF4, a transcription factor, initially induces pro-survival miR-211 and at later it induces pro-apoptotic CHOP. In the current study, we show one microRNA playing a role in ER stress signaling mediated cell death. miR-706 was induced rapidly following ER stress in a PERK and ATF4 dependent manner. Lack of this microRNA suppressed cell death and this phenomenon was reversed, at least in part, by knocking-down of CAAP1. CAAP1 has been demonstrated to be an anti-apoptotic protein (37). Our finding provides further molecular evidence for the ER-stress causing cell death.

miR-706 protects oxidative stress induced hepatic fibrogenesis through blocking PKCα/TAOK1 signaling (38). Lian et al. (39) reported that miR-706 inactivates caspase-3 and caspase-9 and thus inhibit apoptosis induced by vesicular stomatitis virus. However, its role in UPR has never been reported. Our work, for the first time, implies the necessity of PERK/ATF4/ miR-706/ CAAP1 axis in ER stress induced cell death.

Blast search with mmu-miR-706 showed that it is closely matched with Homo sapiens uncharacterized LOC105372576. In future, role of this microRNA in human physiology in the context of ER stress could be investigated.

## Conclusion

Our results identified the fundamental role for miR-706 in regulating cell death induced by ER stress and suggested that miR-706 might be a novel therapeutic target for human cancers. We also provided evidence that CAAP1 is the direct target of miR-706 in response to PERK/AFT4 pathway mediated ER stress.
